# Thermophilic microbial agents promote the fermentation progression of spent mushroom compost and pig manure

**DOI:** 10.3389/fmicb.2025.1575397

**Published:** 2025-06-18

**Authors:** Hongbo Du, Chongchong Lu, Muhanmad Zunair Latif, Jianfeng Du, Yong Liu, Hongxin Li, Xinhua Ding

**Affiliations:** ^1^College of Forestry, Shandong Agricultural University, Tai'an, China; ^2^State Key Laboratory of Wheat Improvement, College of Plant Protection, Shandong Agricultural University, Tai’an, China; ^3^College of Resources and Environment, Henan Institute of Science and Technology, Xinxiang, China; ^4^Shandong Huayang Pesticide Chemical Group Co., Ltd., Tai’an, China; ^5^Jinan Tianding Ecological Environment Co., Ltd., Changqing, China

**Keywords:** antibiotic resistance genes, aerobic fermentation, human pathogenic bacteria, spent mushroom compost, thermophilic microbial agents

## Abstract

Livestock and poultry manure, as a significant organic resource, had an enormous annual production but a utilization rate of less than 50%. Improperly managed manure had become the primary source of agricultural non-point pollution, posing severe challenges to the ecological environment. Achieving efficient resource utilization of livestock manure was a critical step in promoting green agricultural development. Existing research indicated that microbial activity significantly influences the transfer and dissemination of antibiotic resistance genes (ARGs) and the community dynamics of human pathogenic bacteria (HPB) during pig manure composting. However, the specific mechanisms remain unclear. This study innovatively introduced two thermophilic microbial agents (TMS1 and CTMS2) into a pig manure-spent mushroom compost (SMC) aerobic composting system to systematically investigate their regulatory effects on pollutant reduction. The results showed that persistent ARGs (*ErmF*, *ErmQ*, *ErmX*, *blaR1*, *QnrA1*, *QnrA6*, *bla-F*, *QnrA2*, *QnrA5*, *Qnra4* and *bla-VIM*) primarily rely on vertical gene transfer (VGT) for dissemination, whereas easily removable ARGs (*tetX*, *tetW*, *tetG*, *tetC*, *suI1* and *suI2*) were regulated by both horizontal gene transfer (HGT) and VGT. Notably, the co-addition of thermophilic microbial agents and SMC reduced persistent ARGs by lg0.45–3.73, significantly decreased the abundances of HPB such as *Bacteroides* and *Treponema*, and reduced the enrichment of related metabolic pathways, greatly improving compost quality. In stark contrast, the control group (with only SMC and no thermophilic microbial agents) exhibited ARG proliferation. Overall, the application of thermophilic microbial agents not only extended the high temperature phase of composting by over 30% and shortened the composting cycle by 50%, but more importantly, it achieved comprehensive improvement in compost quality by selectively enriching functional microbial communities such as *Pseudomonas*. This study provides a theoretical foundation and data support for the industrial application of CTMS2 in the safe production of organic fertilizers and the synergistic control of environmental risks.

## 1 Introduction

According to statistics, the annual amount of livestock and poultry manure resources in China reaches 4 billion tonnes, of which 40% remain untreated and unutilized, making it the primary source of agricultural non-point source pollution. Among them, pig manure accounts for 36.71% of the total output (He et al., 2021; Zhang S. et al., 2025). Pig manure, an organic complex that riched in crude protein, fiber, and hemifiber (Samanta et al., 2022), could serve as a valuable nitrogen source and slow-release fertilizer. It could enhance soil fertility and improved the physical and chemical environmental properties of soil. However, owing to various technological and process limitations, nearly 60% of pig manure resources was wasted (Wu et al., 2020), posing a great threat to environmental safety. Therefore, exploration of green treatment methods for pig manure and the development of efficient recycling systems for its utilization were urgently needed.

Current treatment methods include anaerobic digestion (AD) and aerobic fermentation (AF). While AD of livestock and poultry manure 76.5% of cellulose and 84.9% of hemicellulose were converted into methane (Ma et al., 2021; Muhammad and Birgitte, 2021), its resilience on specialized equipment and risks of secondary pollution limit practicality. In contrast, AF is simpler, cost–effective, and increasingly adopted for manure treatment (Zhao et al., 2024). Key factors influencing AF efficiency include carbon–to–nitrogen (C/N) ratios, feedstock particle size, and moisture content (Ji et al., 2022), with the optimal performance achieved at a C/N ratios of 25 and a turning frequency of twice per day (Chen et al., 2023). Agricultural wastes like spent mushroom compost (SMC)—a byproduct of mushroom cultivation with high nutrient and water-holding capacity—are widely used to adjust compost properties. For instance, adding 15% woody peat to pig manure reduces nitrogen loss by suppressing denitrifying bacteria and related functional genes (Xie et al., 2023; Wu et al., 2023).

Similarly, co-composting organic waste of different sizes, such as 5 cm corn straw (Ren et al., 2023) and 2 cm branch piles (Zhang D. et al., 2023; Jiao J. X. et al., 2023) with animal manure can reduce greenhouse gas emissions and accelerate the composting process. The co-addition of organic waste during composting has also been shown to inhibit and reduce the expression of antibiotic resistance genes (ARGs), affecting species diversity and ARG migration pathways (Zhou Y. W. et al., 2022). For example, coconut shell, bamboo (Awasthi et al., 2021), wine grape pomace (Zhang J. et al., 2023), and 5% humic acid (Shi et al., 2023) have been reported to enhance antibiotic removal from pig manure and inhibit the accumulation and spread of ARGs (Tong et al., 2022). SMC, a type of agricultural waste, with high water-holding capacity and nutrient content (Tao et al., 2022), had a production volume of 2.2 × 10^7^ tonnes (dry weight) in China in 2020 (Guo et al., 2022). When mixed with chicken manure, it can shorten the high-temperature composting period by 2 days (Pan et al., 2023; Jia et al., 2022), promote humification, and immobilize heavy metals such as Cu, Zn, Cd, Cr, and Pb (Kong et al., 2022). This mixture also reduces emissions of ammonia (NH_3_), hydrogen sulfide (H_2_S), dimethyl sulfur, and dimethyl disulfide (Wei et al., 2022; Yan et al., 2020), recruits beneficial microbial communities, suppresses potential plant pathogens (Xu M. Y. et al., 2022; Wang L. et al., 2024), and significantly reduces the abundances of pathogenic fungi associated with rice blast disease (Zeng et al., 2023).

Microorganisms play an important role in the transformation of organic materials during composting, leading to significant changes in bacterial community composition (Wang Y. et al., 2024). Solid and semi-solid microbial agents, including lignocellulosic hemicellulose-degrading biological agents, thermophilic microbial agents, fungal agents, and antibiotic-degrading agents, contribute to organic matter degradation and nutrient enrichment. These microbial agents also enhance pollutant degradation, alter microbial communities, increase enzyme activity, promote fungal abundance, and immobilize heavy metals (Yin Y. N. et al., 2023; Wu et al., 2022). Additionally, they facilitate ARG removal (Chen X. J. et al., 2022; Li et al., 2022), enhance lignocellulose degradation efficiency (Bikram et al., 2021; Shangguan et al., 2022; Zhang Y. G. et al., 2023), improve methane production rates in AD systems (Bikram et al., 2020), and accelerate substance transformation (Bohrer et al., 2023). Thermophilic microbial agents have been shown to promote the decomposition of recalcitrant organic compounds in biogas residues and improve the seed germination index (Xu S, Y. et al., 2022). In this study, two thermophilic strains, *Bacillus flexus* FM and *B.cereus* KU, were screened and used as microbial agents to study their corresponding effects on promoting pig manure composting fermentation.

Recent research had addressing antibiotic residues in pig manure composting has predominantly examined the effects of non-biological and biological factors on ARGs, cadmium, human pathogenic bacteria (HPB), and other toxic substances in compost materials (Chen Z. Q. et al., 2022; Abdellah et al., 2023; Jiao J. X. et al., 2023). Under heat stress conditions, the abundances of ARGs and mobile genetic elements (MGEs) had been decreasd in pig manure significantly (Sun et al., 2021; Sun et al., 2023; Zhu et al., 2023; Tang et al., 2023). The transmission and transfer of ARGs occured through horizontal gene transfer (HGT) mechanism mediated by MGEs or through vertical gene transfer (VGT) mechanism mediated by host bacterial proliferation and functional gene enrichment (Luo et al., 2023). This study utilized thermophilic microbial agents to promote the co-composting of pig manure and SMC. The research systematically examined the dynamics of ARGs, HPB and the structural and compositional shifts in beneficial microbial communities during composting. The results provided critical theoretical and empirical support for tracking the fate of hazardous contaminants in livestock manure, while advancing the sustainable utilization of livestock and poultry manure and the development of eco-circular agriculture.

## 2 Materials and methods

### 2.1 Screening of thermophilic strains

A total of 62 culturable strains were isolated from pig manure at a farm in Tai’an city, Shandong province, China (Huang et al., 2014; Du et al., 2022). The primary selection criteria were the ability to survive at a high temperature of 60°C, along with the capacity to produce at least two of the following enzymes: cellulase (Subhojit et al., 2016; Warasirin et al., 2017), laccase (Aslam et al., 2012) and xylanase (Fatma and Filiz, 2023). Based on these criteria, B. flexus FM and *B. subtilis* KU were isolated. The fermentation broth of *B. flexus* FM was mixed with soybean meal in a 1:1 ratio to obtain the thermophilic microbial agents S1 (TM S1). Similarly, a composite thermophilic microbial agent S2 (CTM S2) was prepared by mixing the fermentation broths of *B. flexus* and *B. subtilis* KU with soybean meal in a 1:1:2.

### 2.2 Composting experimental design and sample collection

Pig manure was collected from a breeding farm in Tai’an city, Shandong province, China. SMC and pig manure were purchased from Shandong Hengxin Biotechnology Co., Ltd., and Wenshi Pig Breeding Co., Ltd., both of which were located in Shandong province, China. The pig manure had a moisture content of 83.40%, a pH of 7.51, a total carbon content of 30.41%, and a total nitrogen content of 1.74%. The SMC had a moisture content of 62.5%, a pH of 6.39, a total carbon content of 38.57%, and a total nitrogen content of 2.50%.

Four experimental treatments were established: (A) pig manure and TM S1, (B) pig manure and SMC, (C) pig manure, SMC and TMS1, and (D) pig manure, SMC and CTMS2, each treatment was replicated three times. Physicochemical indicators, resistance gene abundance, microbial diversity, and cadmium content were measured on days 0, 1, 4, 7, and 11. Additionally, non-target metabolite indicators were assessed using LC–MS for each treatment group at 0, 4 and 11 days (the B0 sample data were the same as those of the C0 and D0 samples). Each test was performed in triplicate.

### 2.3 Determination of non-biological indicators

Stack and ambient temperatures were recorded three times daily and the average values were calculated. The total carbon (TC) and total nitrogen (TN) contents in the stack were analyzed using an elemental analyzer (Vario Macro Cube, Elementar, Germany). Phosphorus was determined via chromatography (Chen, 2015), while potassium content was measured using tetraphenylboron sodium mass method (Wang et al., 2016), The water content was assessed using the vacuum oven method (Yin Y. Y. et al., 2023), and the cadmium content was determined by atomic fluorescence photometry (Zhang et al., 2015). Each test was conducted also in triplicate.

### 2.4 DNA extraction and qPCR

DNA extraction: Genomic DNA was extracted from 100 mg of freeze-dried samples using the TIANAMP Soil DNA Kit (DP336) and eluted with low melting point solvent (DES). The quality and concentration of the extracted DNA were assessed using 1.5% (w/v) agarose gel electrophoresis and an enzyme plate instrument (BiotekElx808). High-throughput quantitative PCR (HT-qPCR) was performed using a StepOnePlus™ Real-time PCR system (Thermo Fisher Scientific) with a TB Green™ Premium Ex Taq™II (Tli RNaseH Plus) kit (Takara, Code No. RR820A). Each HT-qPCRs assay was conducted in triplicate as described by Gong C. P. et al. (2024).

This study focused on six representative ARGs: (1) tetracycline resistance genes (*tetC*, *tetG*, *tetM*, *tetW* and *tetX*), (2) sulfonamide resistance genes (*sul1* and *sul2*), (3) macrolide resistance genes (*ermF*, *ErmQ* and *ermX*), (4) quinolone resistance genes (*gryA* and *qnrA*), (5) β-lactam resistance genes (bla-VTM and *bla-CTX*), and (6) aminoglycoside genes [*aac (6′)-Ib-cr*]. Additionally, three MGEs, nameed *Tn916/1545*, *intI1*, and *ISCR1*, were analyzed alongwith 16S rRNA for simultaneous quantification (Zhou et al., 2021).

### 2.5 Metagenomic sequencing

Total genomic DNA was extracted from the compost samples using the E.Z.N.A.® Soil DNA Kit (Omega Biotek, Norcross, GA, U.S.) according the manufacturer’s instructions. The concentration and purity of the extracted DNA were measured using a TBS-380 fluorometer and NanoDrop2000 spectrophotometer. The quality of the extracted DNA was verified by electrophoresis on a 1% agarose gel.

Metagenomic data were assembled using MEGAHIT (Li et al., 2015; https://github.com/voutcn/megahit, version 1.1.2), which used succinct de Bruijn graphs. Contigs with a length ≥ 300 bp were selected as the final assembly result, and these contigs were subsequently used for gene prediction and annotation.

Open reading frames (ORFs) from each assembled contig were predicted using MetaGene (Noguchi et al., 2006; http://metagene.cb.k.u-tokyo.ac.jp/). Predicted ORFs with a length of ≥ 100 bp were retrieved and translated into amino acid sequences using the NCBI translation table.^1^ Antibiotic resistance annotation was performed using Diamond (Buchfink et al., 2015; http://www.diamondsearch.org/index.php, version 0.8.35) against the ARDB database^2^ or the CARD database^3^ with an e-value cutoff of 1e^−5^.

### 2.6 Non-target metabolite determination

A 50 mg solid sample was added to a 2 ml centrifuge tube along with a 6 mm diameter grinding bead, and 400 μl of extraction solution (methanol:water = 4:1, v/v) containing 0.02 mg/mL internal standard (L-2-chlorophenylalanine) was added. The samples were then ground using a Wonbio −96°C frozen tissue grinder (Shanghai Wanbo Biotechnology Co., Ltd) for 6 min at −10°C and 50 Hz followed by low-temperature ultrasonic extraction for 30 min at 5°C and 40 kHz. Afterward, the samples were incubated at −20°C for 30 min and centrifuged for 15 min at 4°C and 13,000 rpm, after which the resulting supernatant was transferred to an injection vial for LC–MS analysis.

### 2.7 Statistical analysis

SPSS 25.0 (IBM, Armonk, NY, USA) was used to compare differences and determine correlations among the experimental results. The histograms were generated using Origin 2024b software. Principal coordinate analysis and Procrustes analysis were performed using the Meiji Biocloud platform (Shanghai, China). Heatmap and cluster analysis were conducted with SciPy (Python) Version 1.0.0, whereas differentially abundant metabolite analyses were performed using Ropls (R packages) Version 1.6.2. The correlation coefficient between Spearman and Gephi in the network analysis was determined to be 0.9.2 (*p* < 0.01). AMOS 26.0 software was used for structural equation modeling to elucidate causal relationships between variables.

## 3 Results and discussion

### 3.1 Target ARG and MGE fate during composting process

**Figure 1:**
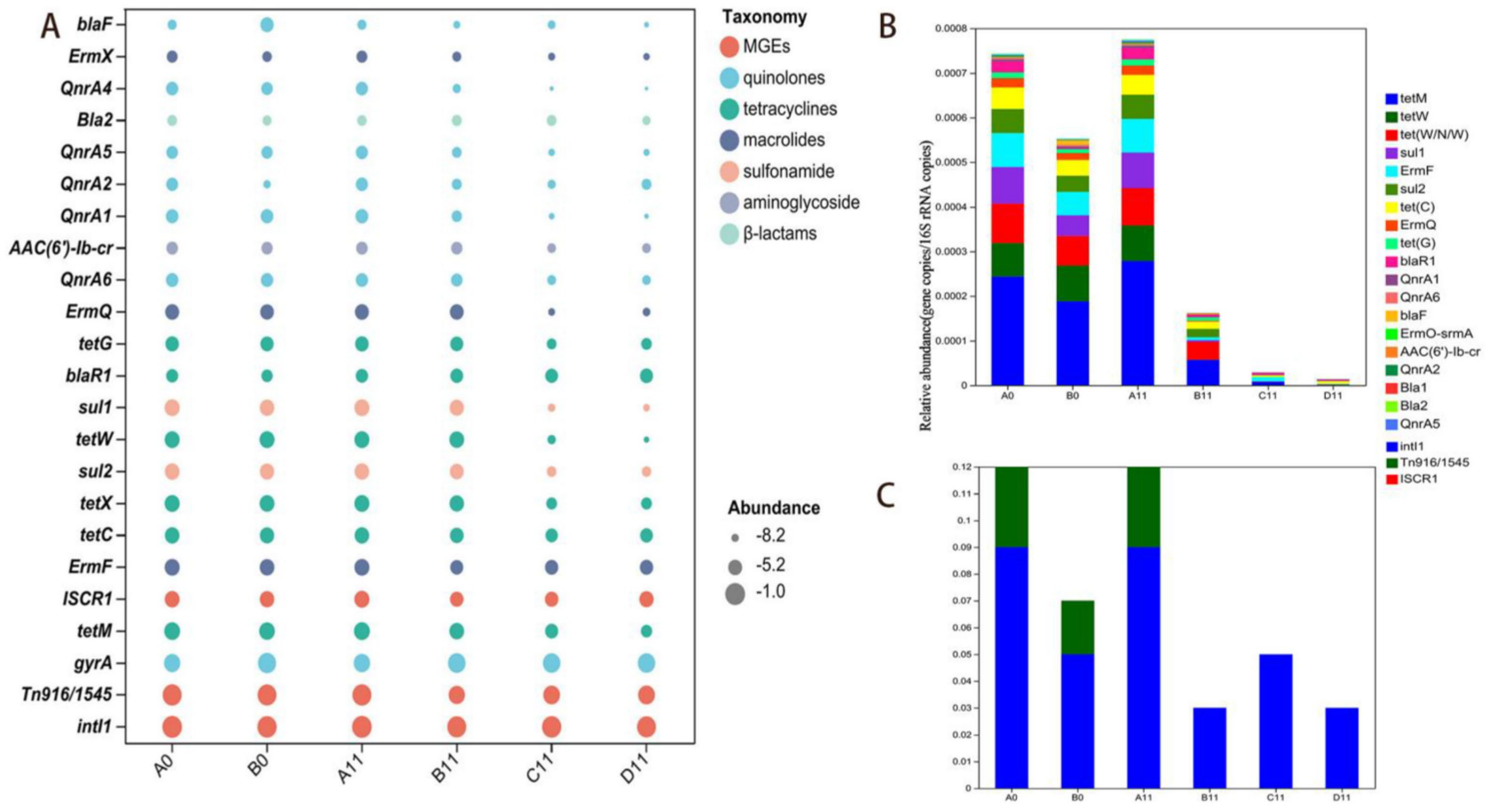
Abundances of ARGs and MGEs at the beginning and end of composting. **(A)** Normalized relative abundance of ARGs and MGEs. The same color indicates that ARGs or MGEs belong to the same category, and the size of the circles indicates the normalized value; **(B)** Accumulated relative abundance of ARGs; **(C)** Accumulated relative abundance of MGEs.

Normalizing the copy number of ARGs to the relative abundances evaluation method of bacterial abundance changes was a more effective way for evaluating ARGs dynamics (Czekalski et al., 2015). In this study, the relative abundances indicators of 15 ARGs and three MGEs had shown that tetracycline, macrolide, sulfonamide, and quinolone-resistant ARGs dominated across all composting samples (Figure 1), Co-composting of livestock manure with plant-derived organic waste effectively reduced the relative abundances of most tetracycline ARGs (*tetM*, *tetW* and *tetX*), sulfonamide ARGs (*sul1* and *sul2*) and macrolide ARGs (*ermF*).

In this study, the addition of thermophilic microbial agents had significantly reduced the abundances of most ARGs in the windrows. Compared with traditional composting methods (Wu et al., 2023; Jiao J. X. et al., 2023), the thermophilic microbial agents extended the high-temperature phase by over 30% and shortened the compost maturation time by 50%. Moreover, compared to B0 sample, the relative abundances of the seven types of ARGs of C11 and D11 samples (*tetM*, *tetW*, *ermF*, *ermQ*, *tetC*, *gryA* and *tetX*) decreased significantly (*p* < 0.05), with values ranging from 0.45–2.98lg and 0.48–3.73lg, respectively. Moreover, compared with those of the C11 sample (11th-day data of C sample), two types of D11 sample (11th-day data of D sample) with ARGs (*tetM* and *tetW*) presented decrease in relative abundances of 0.82lg and 0.74lg, respectively (*p* < 0.05), this indicated that CTMS2 had unique advantages over TMS1 in the degradation of some ARGs. However, compared with those in B0 sample, the two ARGs (*blaR1* and *QnrA2*) in the C11 and D11 samples increased by 0.02–0.63lg and 0.78–0.83lg, respectively, which indicated that removing some heat-resistant ARGs during composting were difficult.

In this study, *IntI1* and *Tn916/1545* emerged as the main constituents contributing to the abundances of MGEs during the composting process (Figure 1C). Compared to those of the B0 sample, the relative abundances of two types of MGEs (*ISCR1* and *Tn916/1545*) had been decreased between 0.08lg and 1.41lg in C11 and D11 samples, highlighted that the efficacy of thermophilic microbial agents in promoting the degradation of MGEs in SMC and pig manure co-composting, which was consistent with trend changes in seven ARGs (*tetM*, *tetW*, *ermF*, *ermQ*, *tetC*, *gryA* and *tetX*). Moreover, compared with that in the C11 sample, the MGE (*intI1*) in the D11 sample had been decreased by 0.28lg (*p* < 0.05), indicated that CTMS2 might have a greater advantage than TMS1 in inhibiting the rebound of MGE abundance.

### 3.2 Analysis of the bacterial community composition structure

The bacterial community was the main driving factor for changes in the composition and abundance of ARGs (Zhang Y. G. et al., 2023), and the differences in bacterial community structure in this study were using principal coordinate analysis to compared and analyzed, as detailed in the Supplementary materials. The results had shown that different samples had a significant effect on the type and abundance of microbial communities in the compost (Supplementary material S6). The interpretation rate of the first and second main components for the results was 85.00%. In addition to the A0 and A11 samples, different samples were clustered together at each stage of compost, indicated that the composition of the bacterial community had an important influence on the progress of pig manure compost (Jie et al., 2023).

**Figure 2:**
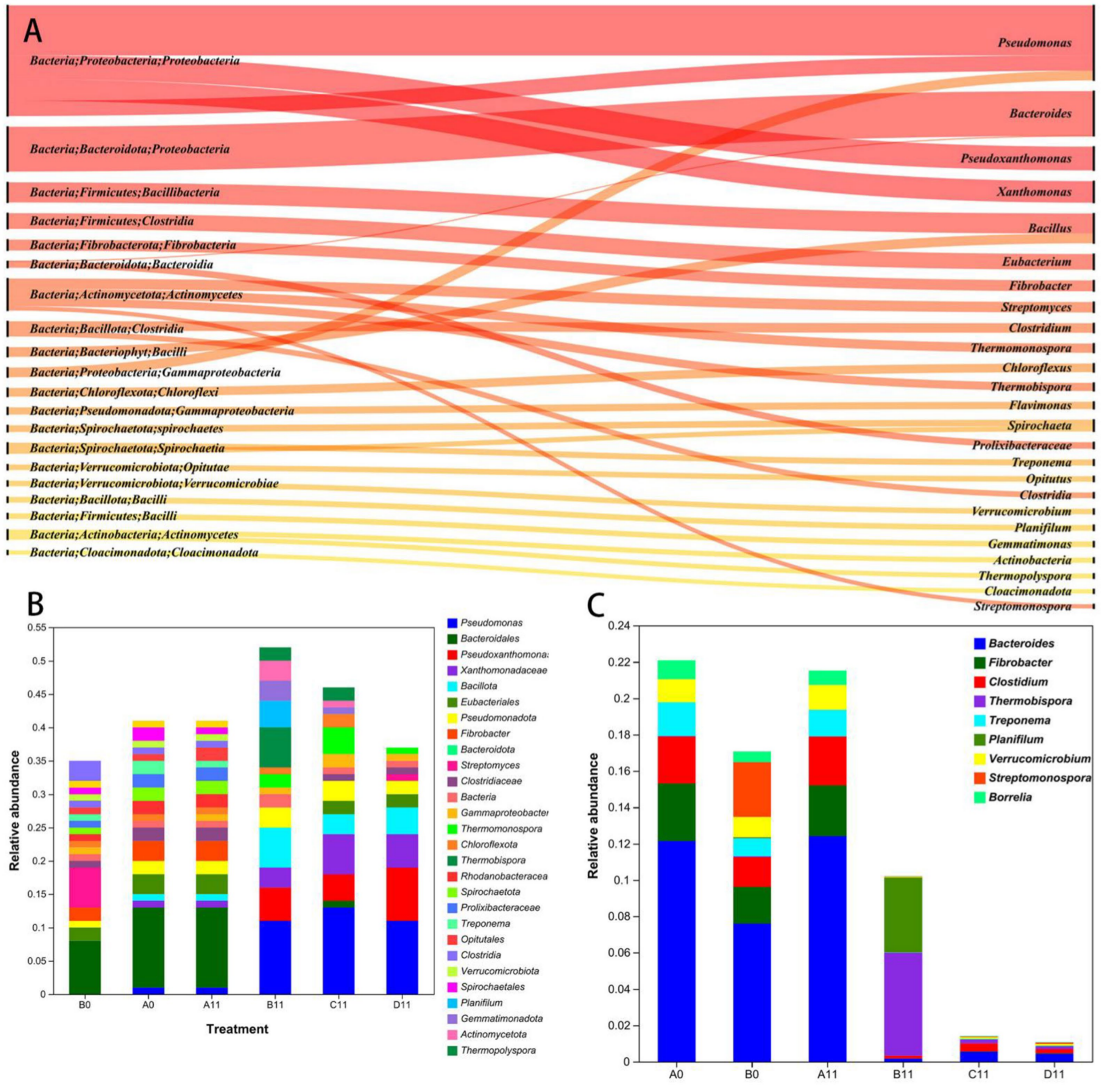
**(A)** Sangi diagram showing the composition of bacterial communities at the phylum, class, order, and genus levels. **(B)** Stacked bar chart showing the relative abundance changes in the top 30 genera of bacteria. **(C)** Stacked bar chart of the abundance of human pathogenic bacteria at the beginning and end of composting.

Figure 2 illustrates the 30 bacterial dominant phyla, classes and genera with the greatest relative abundances during pig manure composting. Among them, the horizontal distribution of the dominant bacterial phyla in the initial stage of pig manure composting was mainly Pseudomonadota, Bacillota, Bacteroidota and Actinomycetota, accounting for more than 62.7% of the total bacterial count in each sample. Moreover, in the high-temperature thermophilic phase of pig manure composting, compared with that in the B0 sample, the abundance of Pseudomonas increased between 1.43lg and 1.37lg in the C11 and D11 samples, whereas the abundance of Bacillota increased between 0.93lg and 0.96lg. Conversely, the abundance of Bacteroidota decreased between 1.15lg and 1.24lg, whereas the abundance of Actinomycetota increased between 1.36lg and 0.89lg in the same sample. Pseudomonadota and Bacillota maintained dominance across all the samples, followed by Actinomycetota. Compared with the B11 sample, the C11 and D11 samples resulted in decreases of 0.60lg and 1.07lg, in Actinomycetota. Therefore, a high level of HGT mechanism might still have occured in sample B and indirectly led to an increase in the abundances of some ARGs.

The 30 most abundant bacteria in the C11 sample were *Pseudomonas* (14.15%), *Xanthomonas* (6.48%) and *Pseudoxanthomonas* (4.87%), whereas those in the sample D11 included *Pseudomonas* (12.23%), *Pseudoxanthomonas* (8.87%) and *Xanthomonas* (5.8%). Compared with those in the B0 sample, *Bacteroides* (9.11%), *Bacillus* (0.47%) and *Actinomyces* (0.03%), significantly changed the abundances of *Bacteroides* (0.59 and 0.67%), *Bacillus* (3.65 and 3.97%), and *Actinomycetes* (0.76 and 0.25%), were observed in the C11 and D11 samples, respectively (see Figure 2B). The increase in the horizontal abundance of *Bacillus* species in the windrow might be due to the increase in the abundance of high-temperature resistant *Bacillus* (see the Supplementary material).

In this study, HPB were found to belong to 9 genera, included *Bacteroides*, *Fibrobacter*, *Clostidium*, and *Trepinema*, which collectively represented 90.91% of the total HPB (Figure 2C). Compared with those in the B0 sample, the HPB abundances in the C11 and D11 samples were 0.82 lg and 0.90 lg lower, respectively (*p* < 0.05), and *Bacteroides* was the pathogenic microorganism with the greatest reduction in abundance, followed by *Treponema*. Most HPB were potential hosts of ARGs and MGEs (Imtiaz et al., 2022), therefore, HPB species and abundances were commonly used as potential measures of ARGs and MGEs. However, the B11 sample, which did not receive the addition of thermophilic microbial agents, exhibited a significant increase in the abundance of *Treponema* (see Supplementary materials). This suggests that some HPB may have undergone incomplete degradation or partial enrichment. These findings further emphasize the necessity of incorporating thermophilic microbial agents in the aerobic composting process of pig manure.

### 3.3 Bacterial community composition and structure lead to changes in ARG abundance

**Figure 3:**
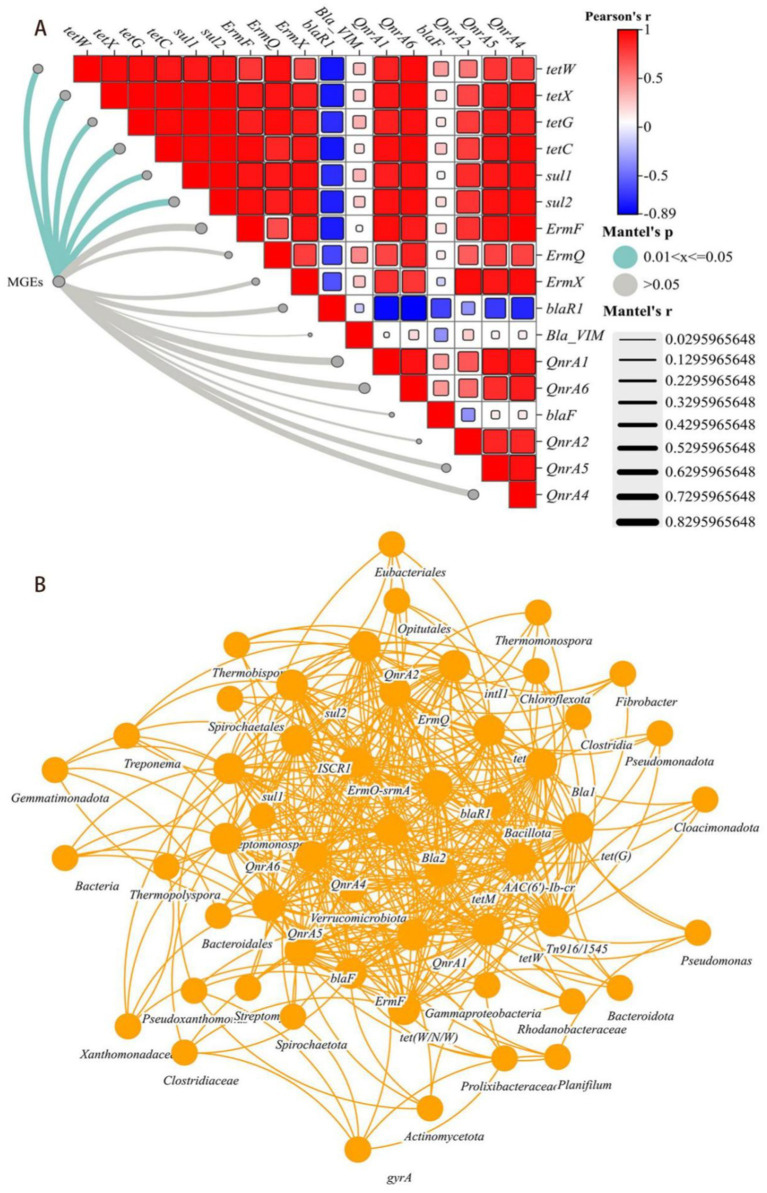
**(A)** Mantel test results based on the correlation between ARGs and MGEs. The color gradient represents the ARGs abundance on the basis of the Spearman correlation coefficient and the edge width and color represent the r-value and statistical significance of the Mantel test, respectively. **(B)** Based on Spearman correlation coefficient network co-occurrence patterns of ARGs, MGEs and their potential host bacteria (top 30 genera) (*p* < 0.01).

Figure 3A showed the correlation between ARGs and MGEs, one-third of the ARGs (*tetX*, *tetW*, *tetG*, *tetC*, *suI1* and *suI2*) were positively correlated with MGEs (*p* < 0.05). However, ARGs which mediated by MGEs could be easily transferred and spreaded after composting, indicated that the inhibitory effect of the HGT mechanism might play a crucial role in preventing their spread. Similarly, the remaining ARGs (*ErmF*, *ErmQ*, *ErmX*, *blaR1*, *QnrA1*, *QnrA6*, *bla-F*, *QnrA2*, *QnrA5*, *Qnra4* and *bla-VIM*) were not significantly correlated with MGEs, and were difficult to degrade during the composting process. Considered that the overall trend of abundance changes during the composting process, its degradation transfer might been mediated by other mechanisms.

Procrustes analysis had revealed that the abundances of ARGs and MGEs were significantly correlated with the bacterial community composition at the genus level (M^2^ = 0.253, *p* < 0.05), which was consistented with the Mantel Test results (r = 0.6062, *p* = 0.01). Many easily removable ARGs (*tetM*, *tetW*, *tetX*, *tetG*, *tetC*, *suI1*, *suI2*, *ermF* and *ermQ*) had been found positively correlated with genus-level changes in multiple bacterial groups (*p* < 0.01), However, persistent removable ARGs correlated with the levels of only a few bacterial genera, such as *Treponema*, *Borrelia* and *Bacteroides*.

### 3.4 Relationships between bacterial microflora and environmental factors

In this study, redundancy analysis (RDA) was used to determine the relative contributions of environmental factors, MGEs and ARGs to the bacterial community, and the explanatory rates of the sample bacterial community were 93.32, 0.70 and 0.27%, respectively (see Supplementary materials). Among these factors, the bacterial community composition had been found to be the main environmental factor. Among all abiotic environmental factors, temperature (99.97%) and TC (99.97%) were accounted for the greatest proportion of bacterial community changes, followed by TK (75.56%). In addition, this study revealed that the changes in ARGs during composting were similar to the changes in bacterial communities and environmental factors as determined by principal coordinate analysis.

**Figure 4:**
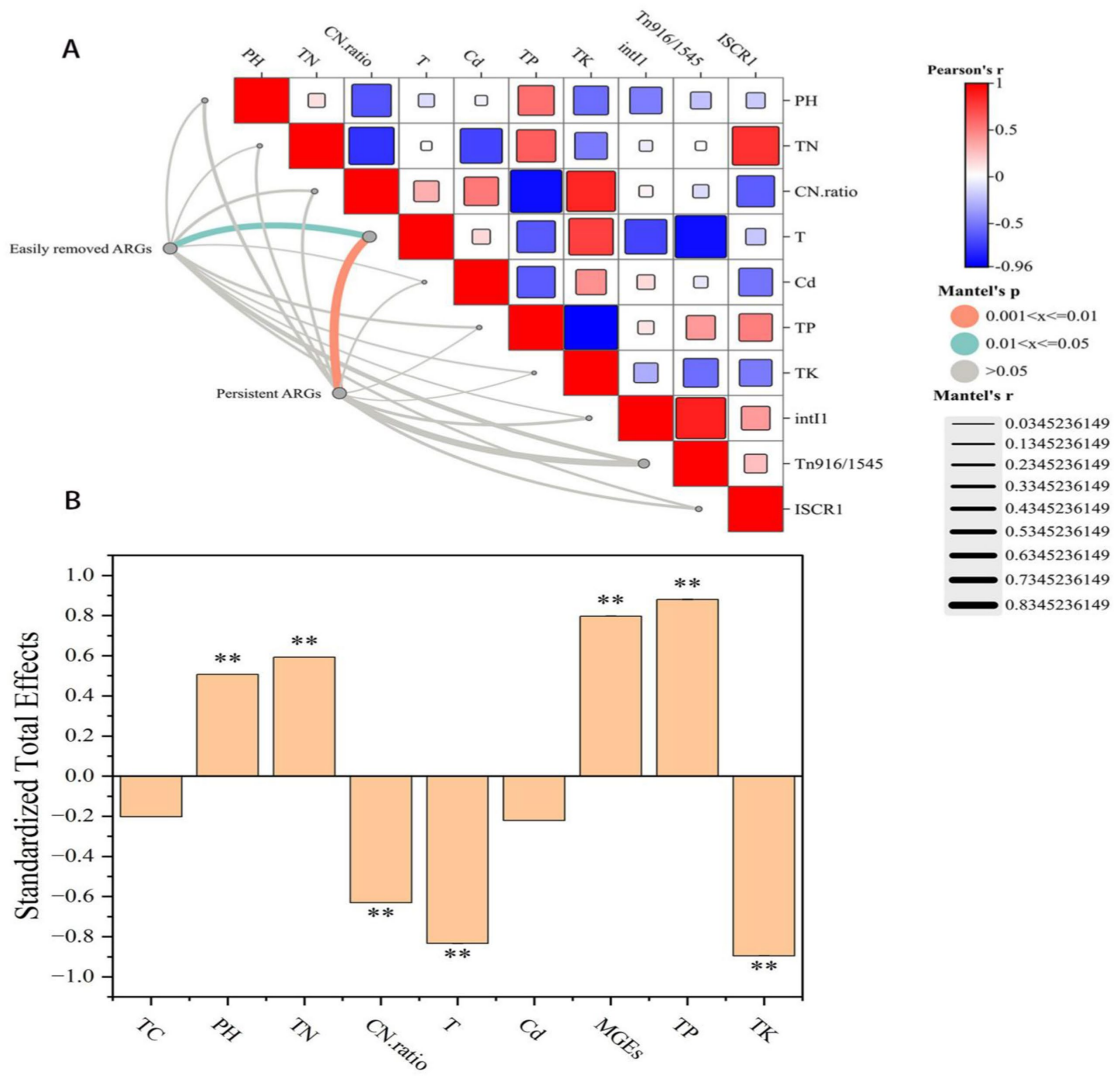
**(A)** Mantel correlation test results between two types of ARGs and multiple indicators (non-biological factors and MGEs). **(B)** Standardized total impact of selected variables of ARGs.

This study used structural equation modeling to explore in more detail the potential causal relationships between multiple factors and ARGs (Figure 4A). The analysis had revealed that compost characteristics had the greatest impact on compost quality (r = 0.413, *p* < 0.01) (Figure 4B), provided important theoretical insights for guide compost in production practices. Temperature also had a negative effect on ARGs, with a relatively high degree of influence (*λ* = −0.834, *p* < 0.01) (Figure 4B), which was consistent with the results of the redundancy analysis. Therefore, temperature was the main driver of changes in the ARG pedigree, the distribution of ARGs during composting might be more closely related to the biological mechanisms of microorganisms.

The organic carbon content in the windrow was significantly negatively correlated with both ARGs (λ = −0.759, *p* < 0.01) and MGEs (λ = −0.538, *p* < 0.01), mainly because of its different effects on microbial communities (Figure 4C). Organic carbon was suspected to be a key influencing factor of the host during composting (r = 0.899, *p* < 0.01), whereas organic carbon was significantly correlated with the quality of the compost (r = 0.303, *p* < 0.01). The addition of SMC that stem from agricultural waste reduced the abundances of *Bacteroides*, *Treponema* and *Borrelia*, which were carriers of persistent ARGs (*sul1*, *tetG* and *bla_VIM*) in the windrow. Moreover, compared with sample A, the addition of SMC had significantly increased the organic carbon content in the samples C11 (17.83%) and D11 (19.71%), which was also a necessary and sufficient condition for normal compost.

Among the non-biological factors, compost characteristics (TN, and C/N ratio) were found strongly correlated with ARGs (λ = −0.624, *p* < 0.01) and MGEs (λ = −0.326, *p* < 0.05) (Figure 4B), which had indirectly affected ARGs through the concentration of organic carbon. According to the RDA results, the C/N ratio had the greatest impact on ARGs. The relatively high nitrogen content and low C/N ratio in the windrow might provide more available nitrogen sources for microbial growth, increasing the TN content in the mature stage of compost (Supplementary materialsS1). Consistent with the results of this study, the difference in nitrogen content is an important environmental factor related to the relative abundance changes of ARGs and MGEs, thermophilic microbial agents were necessary for aerobic compost of pig manure, and the addition of SMC alone could not effectively reduce the abundances of ARGs in the compost.

Interestingly, on the basis of Mantel Analysis, three indicators of microbial nutritional growth (TC content, TN content and the C/N ratio) were found to be common key factors in the metabolism of easily removable ARGs and persistent ARGs. The abundances of both types of ARGs was significantly correlated with temperature (*p* < 0.05) (Figure 4C). Network analysis had revealed that easily removable ARGs could be carried by many potential host bacterial communities and MGEs, whereas persistent ARGs could be carried by only a few possible host bacteria (Figure 3B).

### 3.5 Bacterial microflora and metabolite analysis

**Figure 5:**
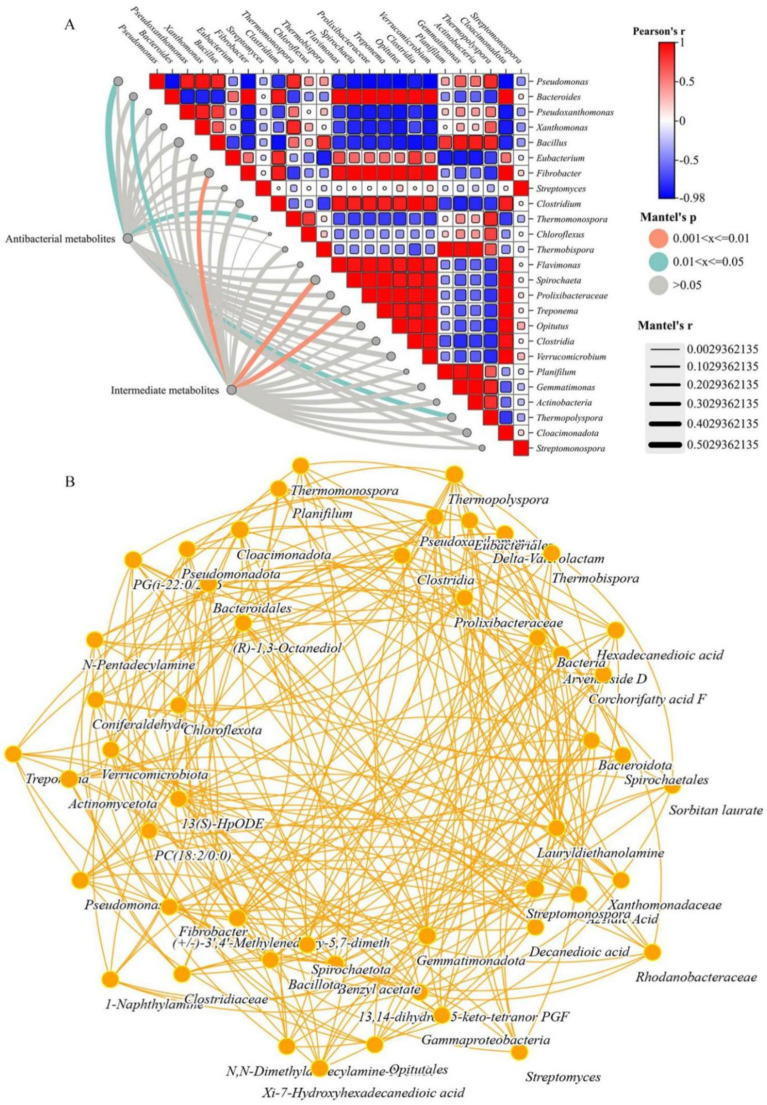
**(A)** Results of the Mantel correlation test between two types of metabolites and the 30 most abundant genera. **(B)** Network co-occurrence patterns of metabolites (20 most abundant) and potential host bacteria (top 30 genera) (*p* < 0.01).

Figure 5A showed the correlation between the 30 most abundant bacterial genera and the 20 most abundant metabolites in the compost. Approximately one-third of the antibacterial metabolites such as corchorifatty acid F, coniferaldehide, 13,14-dihydro-15-keto-tetranor PGF, sorbitan laurate, and PG [i-22:0/22:6 (5Z, 7Z, 10Z, 13Z, 16Z, 1, and azelaic acid)], were associated with the induction of plant resistance metabolism (Masanobu et al., 2020; Leonard et al., 2020). These genera presented significant positive correlations with the abundances of *Pseudomonas*, *Thermomonospora*, and *Thermopolyspora* (*p* < 0.05). This enrichment also indicated that the resistance of the compost material to the growth and infection of pathogenic microorganisms increased (Supplementary materials). Research had shown that high doses of macrolide drugs inhibited the mineralization of natural 17 β-estradiol in animal manure and urine, thereby increasin the retention of 17β-estradiol and its metabolites in free and non-extractable residue forms (He et al., 2019), and the key molecule azelaic acid in the plant biological stress response could mobilize Arabidopsis thaliana immunity in a concentration dependent manner (Francesca et al., 2019; Nagy et al., 2017; Finni et al., 2014). In-depth research and exploration of the relationships between metabolic products in compost and changes in bacterial flora and related functional gene abundance had important practical significance for controlling ARGs transfer.

Microorganisms were the main influencing factors of changes in the composition and content of antimicrobial and intermediate metabolites, so studying and determining their relationships was of great practical significance. Procrustes analysis had shown that antibacterial and intermediate metabolites were significantly correlated with the bacterial community composition at the genus level (M^2^ = 0.253, *p* < 0.01) which was consistent with the Mantel test results (r = 0.5029, *p* = 0.01). Therefore, the changes in bacterial community structure and abundance led to changes in the metabolites detected during the composting process. Furthermore, the co-occurrence associations between specific antibacterial metabolites, bacterial genera, and intermediate metabolites were explored to identify their potential hosts during the composting process. According to the network diagram, there were 6 antibacterial metabolites, 3 intermediate metabolites and 30 bacterial genera (Figure 3B). Differences in the distribution of potential hosts in rhizosphere soils were related to the abundance and composition of metabolites (Guo et al., 2020). Many easily removable antibacterial metals (corchorifatty acid F, coniferaldehide, 13,14-dihydro-15-keto-tetranal PGF, sorbitan laurate, and PG [i-22:0/22:6 (5Z, 7Z, 10Z, 13Z, 16Z, 1, and azelaic acid]) were significantly positively correlated with the abundances of multiple bacterial genera (*p* < 0.05) (Supplementary materials), indirectly indicating that the composition and abundance of bacteria in the windrow and the inhibition of the HGT mechanism might play crucial roles in preventing its spread. Similarly, intermediate metabolites (1-naphthylamine, benzyl acetate and xi-7-hydroxyhexadecanedioic acid) were products of antibiotics, benzene rings and pesticide metabolic intermediates. These genera were significantly positively correlated with the abundances of *Bacteroides*, *Fibrobacter, Treponema* and *Spirochaeta* genera (*p* < 0.01) (Supplementary material), and it was observed that they were difficult to metabolize and degrade during composting, resulting in incomplete or partial enrichment of HPB degradation.

### 3.6 Prediction of bacterial community function

Functional enrichment analysis was a computational method used to analyze the degree of functional pattern enrichment in gene sets or genomic data (Mahantesha et al., 2013). This information could help researchers understand the biological significance of gene sets, thereby revealing the regulatory mechanisms of biological processes, metabolic pathways, cellular components, etc., under specific conditions and providing valuable guidance for further experimental design and research (Zakrzewski et al., 2013).

**Figure 6:**
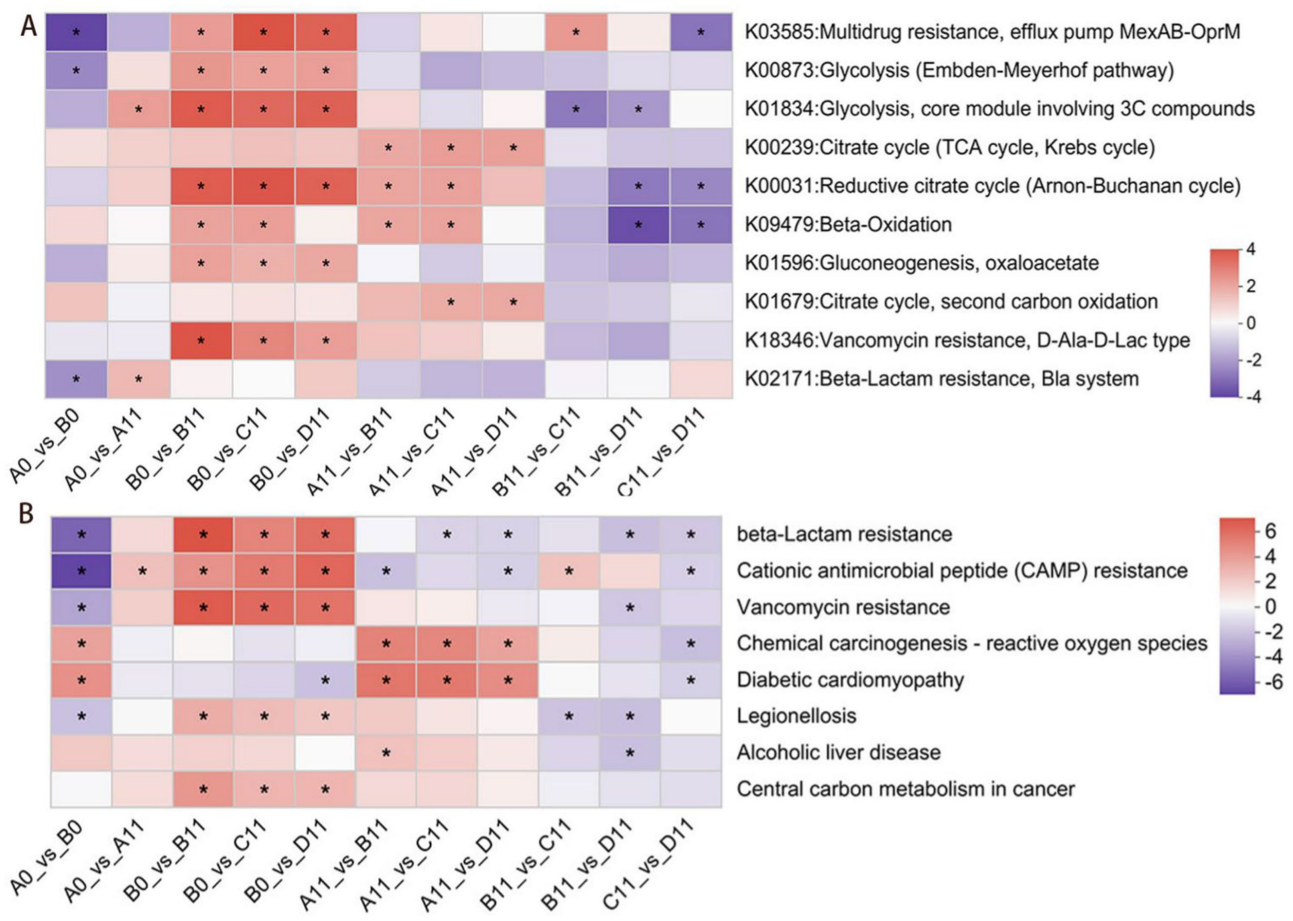
Enrichment plots of human disease-associated metabolic functions and relative abundance of genes under different treatment conditions during composting.A-KEGG enrichment map of the metabolic functions associated with ARGs.B-Human disease KEGG pathway 3 level cluster.

Compared with sample A, the mixed composting of thermophilic microbial agents and SMC with pig manure reduced the metabolic abundance of multidrug resistance efflux pumps, the Embden-Meyerhof pathway and beta-lactam resistance pathway in mature compost piles (Figure 6A). This reduction might promote the metabolism of multiple beta-lactam antibiotics and inhibit the VGT mechanism and the HGT mechanism of ARGs from compost products to the soil environment. The transmission of ARGs was usually regulated by key regulatory genes in certain bacterial pathways. In previous studies, 10 specific genes involved in glycolysis, multidrug-resistance efflux pumps and β-lactam resistance regulation were identified (Lin et al., 2021). As shown in Figure 6A, compared with the A11 sample, the addition of SMC was more effective than the addition of multiple drug resistant efflux pumps. Furthermore, the enrichment of pathways related to the metabolism of multiple antibiotics in β-lactam (K03585 and K02171) was relatively low, indicating that thermophilic microbial agents might inhibit the expression of related genes. Moreover, compared with those in the sample A, the enrichment of ARG-related metabolic pathways had been decreased to varying degrees by the additive of TMS1 (sample C) and CTMS2 (sample D), furthermore, the CTMS2 showed the greatest decrease in the total abundances of ARG-related genes. Therefore, in the context of co-composting SMC and thermophilic microbial agents, the combination of CTMS2 and SMC had a greater inhibitory effect on the expression of ARG-related genes, led to a deeper decrease in the abundances of ARGs (Fu et al., 2024).

Human disease-related KEGG pathway 3 significantly changed during the composting process (Figure 6B). The enrichment of clusters associated with human disease, such as β-lactam resistance, cationic antimicrobial peptide resistance, vancomycin resistance, legionellosis, alcoholic liver disease, and central carbon metabolism in cancer, tended to decrease, with the D sample showing the greatest decrease during composting. Notably, although samples C and D presented a reduced abundance of clusters related to infectious diseases, the residual levels in sample C were significantly greater than those in sample D. Therefore, on basis of the results had shown that in the HPB abundances stacking bar chart (Figure 2C), it could be find that the co-composting process of pig manure utilizing a combination of CTMS2 and SMC was to yield compost products with less harmless compost products than that of sample C.

## 4 Conclusion

The findings of this study had indicated that the co-addition of organic waste such as SMC, which was the basis for the successful compost of pig manure during aerobic composting, and had advantages in reducing the abundances of ARGs, MGEs, and HPB. The co-composting of thermophilic microbial agents and SMC could limit the proliferation of ARG-related hosts and decreas the abundances of ARG-related metabolic pathways, regulatory genes, and human disease clusters, potentially leading to ARG attenuation. The VGT mechanism might play a key role in shaping the progressive degradation of ARGs, especially on persistent ARGs. Compared with TMS1, the CTMS2 had significantly better effects and potential in the compost process, which was convenient for storage, transportation, and easy to use, promoted rapid heating of the compost, had a long duration of high temperature, and was conducive to killing pathogenic bacteria in the compost. In addition, when site requirements were not strict, organic fertilizer could be composted separately by farmers and returned to the field or sold on a large scale, and the organic fertilizer produced had good quality and great application and promotion value.

## 5 Discussion

The misuse of antibiotics in livestock farming and the unregulated discharge of livestock and poultry manure (Li S. Y. et al., 2023), had caused severe environmental pollution while promoting the enrichment and transmission of ARGs to plants and animals, posing significant risks to human health (Zhang Y. et al., 2025; Wu et al., 2025). However, aerobic composting had been demonstrated to substantially reduce antibiotic residues and ARG abundance in livestock manure (Li et al., 2024; Zhao et al., 2024). Research had found that co-composting of livestock and poultry manure with plant-derived organic waste could effectively reduce the relative abundances of most tetracycline ARGs (*tetM, tetW*, *tetX*), sulfonamide ARGs (*sul1* and *sul2*), and macrolide ARGs (*ermF*) (Jiao J. X. et al., 2023; Fu et al., 2024). The abundance of most ARGs were significantly associated with MGEs, and reducing the abundance and suppressing HGT spread of MGEs could mitigate the spread and diffusion of ARGs (Liu et al., 2023). Bacteroidota, Proteobacteria and Actinomycetota were potential hosts associated with the enrichment and transfer of ARGs and MGEs, and were the main carriers for the transmission of ARGs and MGEs (Wang et al., 2022; He et al., 2023; Wu et al., 2024). The solid bacterial agents (TMS1 and CTMS2) inhibited the enrichment and transfer of ARGs and MGEs by altering the composition and abundance of bacteria in the windrow.

Thermophilic composting, as an innovative composting technology involving exogenous thermophilic microbial agents (Zhang Y. G. et al., 2023), induces sustained high temperatures that promoted both the degradation of extracellular ARGs (eARGs) (Jiao J. X. et al., 2023) and the release of intracellular ARGs (iARGs). Certain thermophilic microorganisms, such as *Novibacillus thermophiles*, *Bacillus thermolactis* and *Ammoniibacillus agariperforans*, could accelerate cellulolytic and xylanolytic decomposition. This process enhances microbial diversity in compost materials while facilitating the recruitment of beneficial microbes and suppressing pathogen proliferation (Youn et al., 2020; Wang et al., 2023), thereby optimizing both the composting efficiency and final product quality of livestock manure (Bang et al., 2024; Wang L. et al., 2024). Notably, Zhang et al. (2022) identified significant positive correlations between specific ARGs in swine manure (including *tetC*, *tetG*, *tetX*, *sul1* and *qnrS*) and the abundances of pathogenic microorganisms. During the compost maturation phase, the abundances of pathogenic microorganisms from *Bacteroides* and *Verrucomicrobia* genera progressively decreased, consequently reducing pathogen-mediated VGT of ARGs and significantly enhancing the quality of the organic fertilizer product. While elevated temperatures generally accelerate bacterial community succession, in contrast to conventional microbial inoculants, the TMS1 and CTMS2 that employed in this study exhibited remarkable thermophilic properties, maintaining viability even when compost temperatures exceeded 60°C. Compared to previous composting research, these novel thermophilic microbial agents demonstrated rapid temperature elevation in composting materials, extended duration of the thermophilic phase, and achievement of higher peak temperatures. These superior thermal characteristics collectively contributed to enhanced nutrient preservation, more efficient degradation of hazardous substances, and ultimately, the production of higher quality organic fertilizers.

Research had shown that, ARGs could be distributed through aerobic composting (Ren et al., 2023), anaerobic composting (Chen et al., 2023), soil mediums (Wen et al., 2024) and rivers mediums (Patel et al., 2024). ARGs had a broad range of potential hosts and they could regulate the abundances changes of ARGs by mediating the abundances of related factors such as (tetW, *sul1* and *Tn916/1545*) (Xiu et al., 2021; Zhou et al., 2021; Zhang D. et al., 2023). Moreover, microorganisms were the primary carriers of ARGs and MGEs, therefore, the changes in bacterial community composition and abundance led to changes in the relative abundances of ARGs and MGEs during the composting process (Wang Y. et al., 2024; Nnorom et al., 2025).

In this study, the SMC was co-composting with pig manure, the abundances of some bacterial genera which associated with organic compound degradation increased during the maturation stage, which might have led to a decrease in the abundances of potential host bacteria carrying ARGs and MGEs, at the same time, the proliferation of microbial communities associated with organic matter degradation might limit the growth of some potential hosts of ARGs (Wei et al., 2022; He et al., 2023; Wang et al., 2023). Temperature was considered the most important abiotic factor in aerobic composting processes, and changes in temperature could greatly alter the abundance and content of microbial communities and other abiotic factors (He et al., 2023; Zhao et al., 2024). Although some researchers believed that HGT is one of the important factors for the transfer of ARGs during composting, HGT might not be the main driver of ARGs distribution (Liu et al., 2023; Zhou P. Z. et al., 2022). In other words, this meant that the distribution of ARGs during composting might be more closely related to the biological mechanisms of microorganisms.

In fact, the compost properties were often considered nutrients for bacteria, as most of them were essential for microbial growth (Magid et al., 2006; Grosso et al., 2016), which was potentially relate to the VGT mechanism of ARGs. Some compost characteristics could be adjusted by adding organic carbon to the compost stage, which could affect the structural composition of bacterial communities during the composting process (Gong X. et al., 2024; Hu et al., 2023), which could affect changes in ARGs also (Ya et al., 2023; Shan et al., 2024). Guo et al. (2019) reported that differences in nitrogen content were important environmental factors which related to changes in the relative abundances of ARGs and MGEs. The TC content, TN content and C/N ratio were important environmental factors for the survival and reproduction of microorganisms in livestock and poultry manure, which might indirectly affect the abundances of ARGs and MGEs by affecting changes in the host bacterial community of ARGs (Sun et al., 2020; Kai et al., 2023; Jiao M. N. et al., 2023). Therefore, these findings suggested that the VGT mechanism might play a key role mechanism in the transfer and transformation of ARGs during the composting process in this study, especially for persistent ARGs, whereas easily removable ARGs were also regulated by the HGT mechanism.

## Data Availability

The original contributions presented in the study are included in the article/Supplementary material, further inquiries can be directed to the corresponding author.

## Footnotes

^1^http://www.ncbi. nlm.nih.gov/Taxonomy/taxonomyhome.html/index.cgi?chapter=tgencodes#SG1

^2^ http://ardb.cbcb.umd.edu/

^3^ https://card.mcmaster.ca/home
