# Supplementary material for: Thermophilic microbial agents promote the fermentation progression of spent mushroom compost and pig manure
Source: Front Microbiol. 2025 Jun 18;16:1575397. doi: 10.3389/fmicb.2025.1575397 (PMC12213725; doi:10.3389/fmicb.2025.1575397)
Supplement: Supplementary file 1 [file Data_Sheet_1.docx]

1.Table S1. Alpha-diversity of bacterial community structure during composting

| Treatment | chao | shannon | simpson |
| --- | --- | --- | --- |
| A0 | 28327.67 | 5.31 | 0.03 |
| A11 | 30016.00 | 5.30 | 0.03 |
| B0 | 33928.33 | 5.77 | 0.02 |
| B11 | 31423.00 | 5.37 | 0.02 |
| C11 | 31996.33 | 5.70 | 0.02 |
| D11 | 36343.67 | 5.91 | 0.02 |

1. Table S2. Changes in environmental factor during composting process.

| Treatment | TC  （g/kg) | pH | TN  （g/kg) | CN.ratio | Cd  (mg/kg) | TP  （g/kg) | TK  （g/kg) |
| --- | --- | --- | --- | --- | --- | --- | --- |
| A0_1 | 30.11 | 7.54 | 1.80 | 16.70 | 0.97 | 6.17 | 0.38 |
| A0_2 | 30.46 | 7.48 | 1.76 | 17.34 | 0.93 | 5.95 | 0.36 |
| A0_3 | 30.67 | 7.51 | 1.67 | 18.35 | 0.95 | 5.85 | 0.39 |
| A1_1 | 30.37 | 7.77 | 1.67 | 18.16 | 0.95 | 5.89 | 0.35 |
| A1_2 | 30.38 | 7.71 | 1.67 | 18.22 | 0.91 | 6.27 | 0.35 |
| A1_3 | 30.46 | 7.73 | 1.64 | 18.59 | 0.93 | 6.37 | 0.37 |
| A4_1 | 39.80 | 7.82 | 1.88 | 21.19 | 0.99 | 6.44 | 0.39 |
| A4_2 | 39.54 | 7.84 | 1.93 | 20.47 | 0.96 | 6.42 | 0.38 |
| A4_3 | 39.69 | 7.80 | 2.00 | 19.86 | 0.97 | 6.28 | 0.39 |
| A7_1 | 30.43 | 7.52 | 1.78 | 17.11 | 0.94 | 6.55 | 0.30 |
| A7_2 | 30.11 | 7.68 | 1.84 | 16.40 | 0.91 | 6.26 | 0.32 |
| A7_3 | 30.53 | 7.59 | 1.83 | 16.72 | 0.92 | 6.16 | 0.33 |
| A11_1 | 30.24 | 7.76 | 1.71 | 17.70 | 0.92 | 6.70 | 0.37 |
| A11_2 | 30.91 | 7.71 | 1.73 | 17.84 | 0.89 | 6.57 | 0.35 |
| A11_3 | 30.19 | 7.73 | 1.77 | 17.02 | 0.90 | 6.90 | 0.35 |
| B0_1 | 35.01 | 6.03 | 1.38 | 25.37 | 0.99 | 3.41 | 0.79 |
| B0_2 | 34.49 | 6.00 | 1.37 | 25.10 | 0.93 | 3.55 | 0.86 |
| B0_3 | 35.21 | 6.00 | 1.40 | 25.21 | 0.93 | 3.30 | 0.79 |
| B1_1 | 34.87 | 6.32 | 1.36 | 25.63 | 0.94 | 3.40 | 0.75 |
| B1_2 | 35.07 | 6.51 | 1.40 | 25.01 | 0.91 | 3.17 | 0.75 |
| B1_3 | 35.44 | 6.48 | 1.37 | 25.87 | 0.92 | 3.12 | 0.73 |
| B4_1 | 32.21 | 6.55 | 1.33 | 24.22 | 1.02 | 3.98 | 0.76 |
| B4_2 | 33.51 | 6.59 | 1.38 | 24.30 | 0.98 | 3.64 | 0.73 |
| B4_3 | 31.99 | 6.41 | 1.33 | 24.10 | 0.99 | 3.93 | 0.80 |
| B7_1 | 32.65 | 7.38 | 1.37 | 23.90 | 0.98 | 3.29 | 0.80 |
| B7_2 | 31.67 | 7.24 | 1.34 | 23.71 | 0.94 | 3.18 | 0.77 |
| B7_3 | 32.97 | 7.42 | 1.40 | 23.60 | 0.96 | 3.35 | 0.79 |
| B11_1 | 28.92 | 7.82 | 1.28 | 22.61 | 0.99 | 3.65 | 0.76 |
| B11_2 | 28.95 | 7.86 | 1.28 | 22.70 | 0.95 | 3.81 | 0.78 |
| B11_3 | 29.47 | 7.91 | 1.32 | 22.41 | 0.97 | 3.73 | 0.76 |
| C0_1 | 35.66 | 6.19 | 1.43 | 25.01 | 1.01 | 3.57 | 0.77 |
| C0_2 | 35.09 | 6.19 | 1.40 | 25.01 | 0.97 | 3.54 | 0.71 |
| C0_3 | 35.66 | 6.02 | 1.42 | 25.20 | 0.97 | 3.67 | 0.72 |
| C1_1 | 33.79 | 6.21 | 1.59 | 21.29 | 0.99 | 4.25 | 0.81 |
| C1_2 | 33.36 | 6.12 | 1.58 | 21.14 | 0.96 | 4.59 | 0.86 |
| C1_3 | 33.80 | 6.18 | 1.60 | 21.10 | 0.97 | 4.41 | 0.81 |
| C4_1 | 33.68 | 6.47 | 1.62 | 20.82 | 0.92 | 4.09 | 0.82 |
| C4_2 | 33.82 | 6.33 | 1.68 | 20.16 | 0.89 | 4.05 | 0.85 |
| C4_3 | 33.64 | 6.37 | 1.71 | 19.69 | 0.90 | 3.98 | 0.84 |
| C7_1 | 34.34 | 6.32 | 1.56 | 22.06 | 0.902 | 3.91 | 0.77 |
| C7_2 | 34.42 | 6.33 | 1.56 | 22.02 | 0.87 | 3.90 | 0.78 |
| C7_3 | 34.36 | 6.29 | 1.59 | 21.58 | 0.88 | 3.86 | 0.75 |
| C11_1 | 35.97 | 6.65 | 1.74 | 20.71 | 0.90 | 4.06 | 0.75 |
| C11_2 | 35.83 | 6.51 | 1.69 | 21.20 | 0.91 | 4.20 | 0.76 |
| C11_3 | 35.82 | 6.58 | 1.72 | 20.80 | 0.93 | 3.96 | 0.77 |
| D0_1 | 35.61 | 6.07 | 1.37 | 25.93 | 1.02 | 3.47 | 0.72 |
| D0_2 | 35.68 | 6.05 | 1.42 | 25.17 | 1.00 | 3.52 | 0.75 |
| D0_3 | 35.43 | 6.07 | 1.38 | 25.69 | 1.02 | 3.45 | 0.73 |
| D1_1 | 33.93 | 6.73 | 1.37 | 24.86 | 1.00 | 3.79 | 0.63 |
| D1_2 | 33.74 | 6.91 | 1.39 | 24.24 | 0.99 | 3.92 | 0.62 |
| D1_3 | 33.73 | 6.93 | 1.39 | 24.23 | 0.99 | 3.70 | 0.66 |
| D4_1 | 34.26 | 6.75 | 1.56 | 22.01 | 1.01 | 3.91 | 0.68 |
| D4_2 | 34.12 | 6.56 | 1.56 | 21.91 | 1.00 | 3.87 | 0.71 |
| D4_3 | 34.19 | 6.62 | 1.64 | 20.83 | 0.99 | 3.85 | 0.74 |
| D7_1 | 35.24 | 6.65 | 1.57 | 22.49 | 1.02 | 3.9 | 0.77 |
| D7_2 | 35.22 | 6.68 | 1.57 | 22.50 | 0.98 | 3.91 | 0.78 |
| D7_3 | 35.35 | 6.49 | 1.68 | 21.04 | 0.99 | 4.04 | 0.76 |
| D11_1 | 36.93 | 7.28 | 1.80 | 20.48 | 0.95 | 4.45 | 0.77 |
| D11_2 | 36.02 | 7.26 | 1.76 | 20.50 | 0.92 | 4.22 | 0.77 |
| D11_3 | 36.39 | 7.15 | 1.78 | 20.50 | 0.93 | 4.41 | 0.83 |

1. Figure. S1 Variation of temperature during composting process.


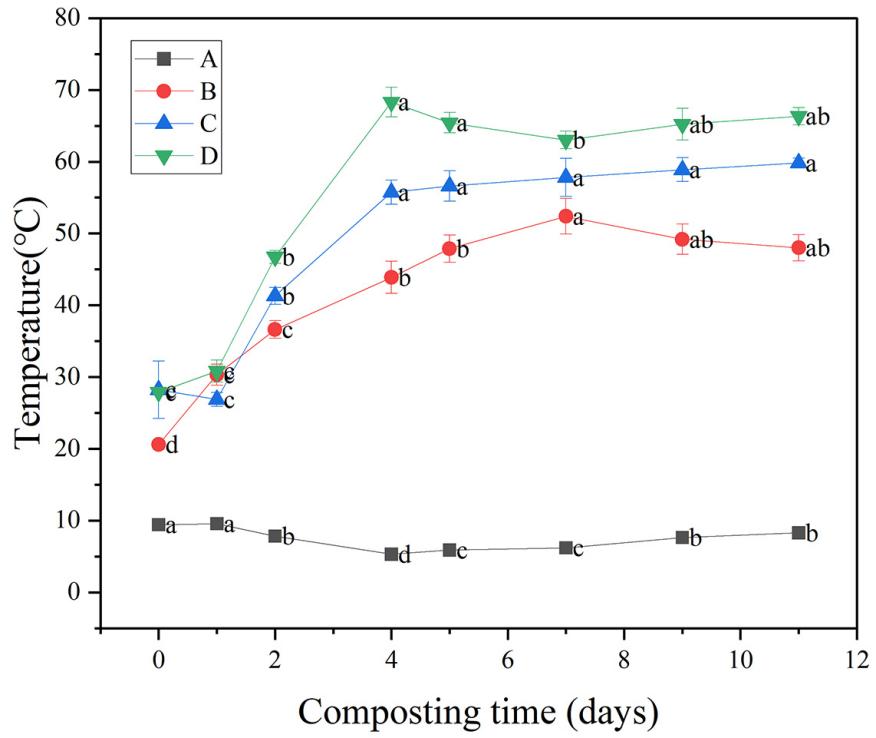


1. Figure. S2 Principal coordinate analysis of the succession of the bacterial communities during composting.


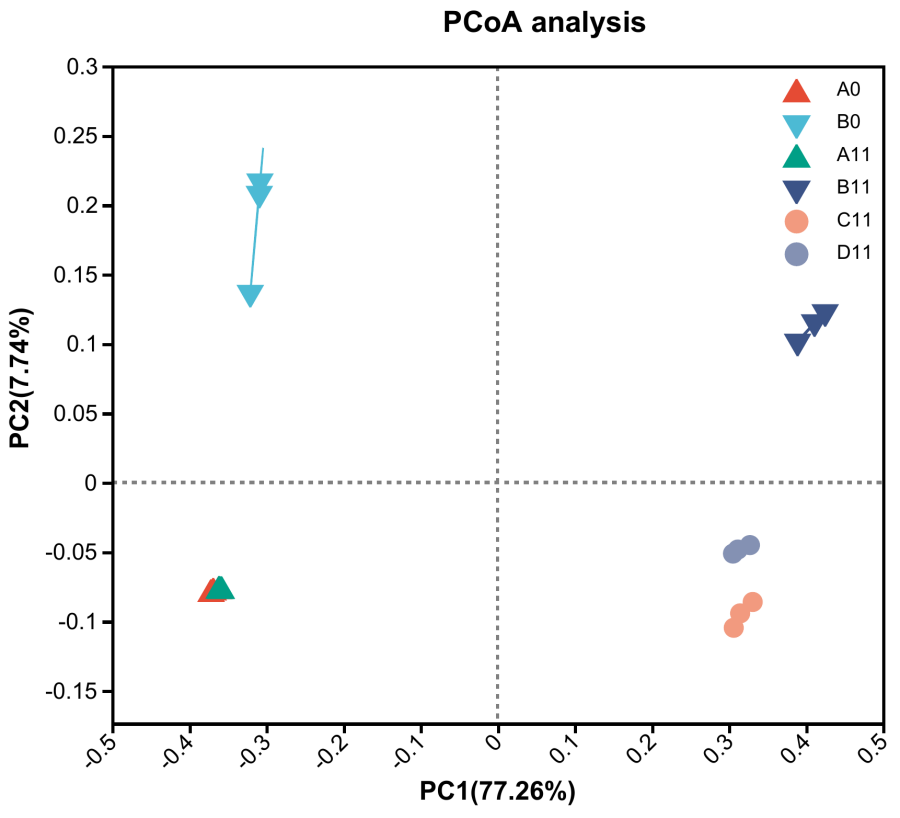


1. Figure. S3 Redundancy analysis to identify the contributions of the bacterial community, abiotic factors, and MGEs to ARGs profiles.


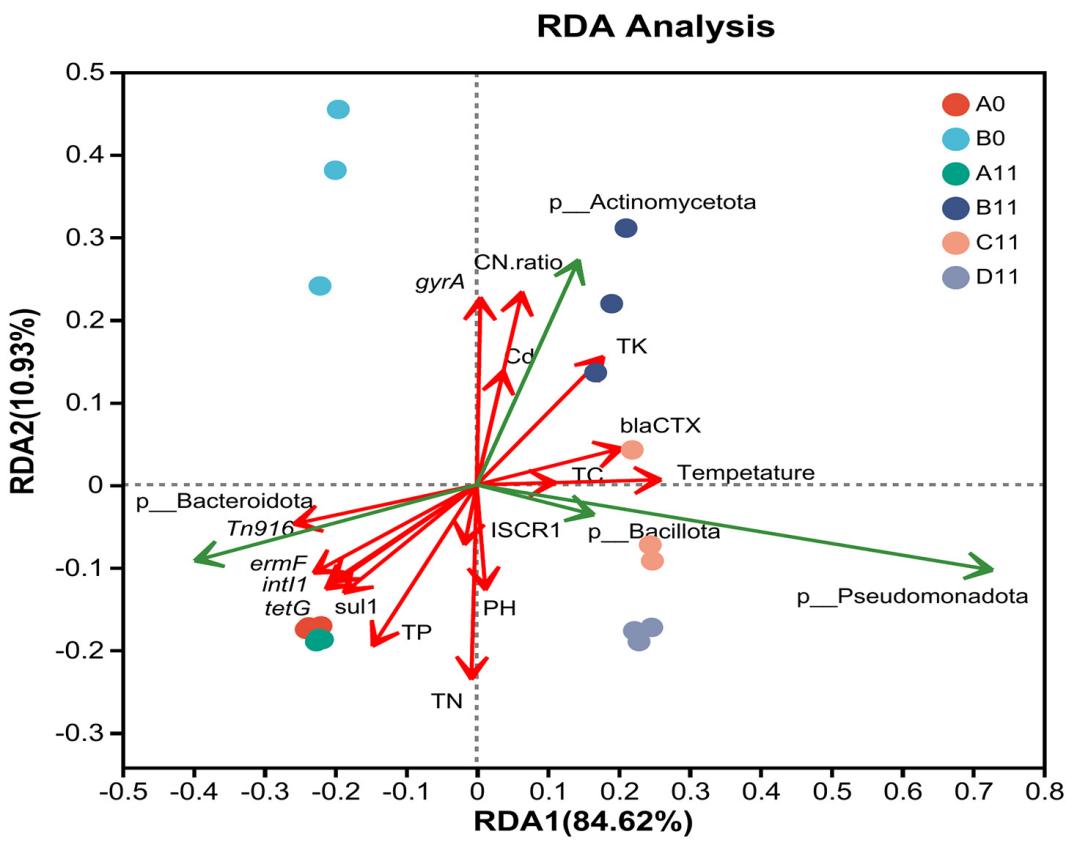


1. Figure. S4 Heat map of microbial metabolism correlation


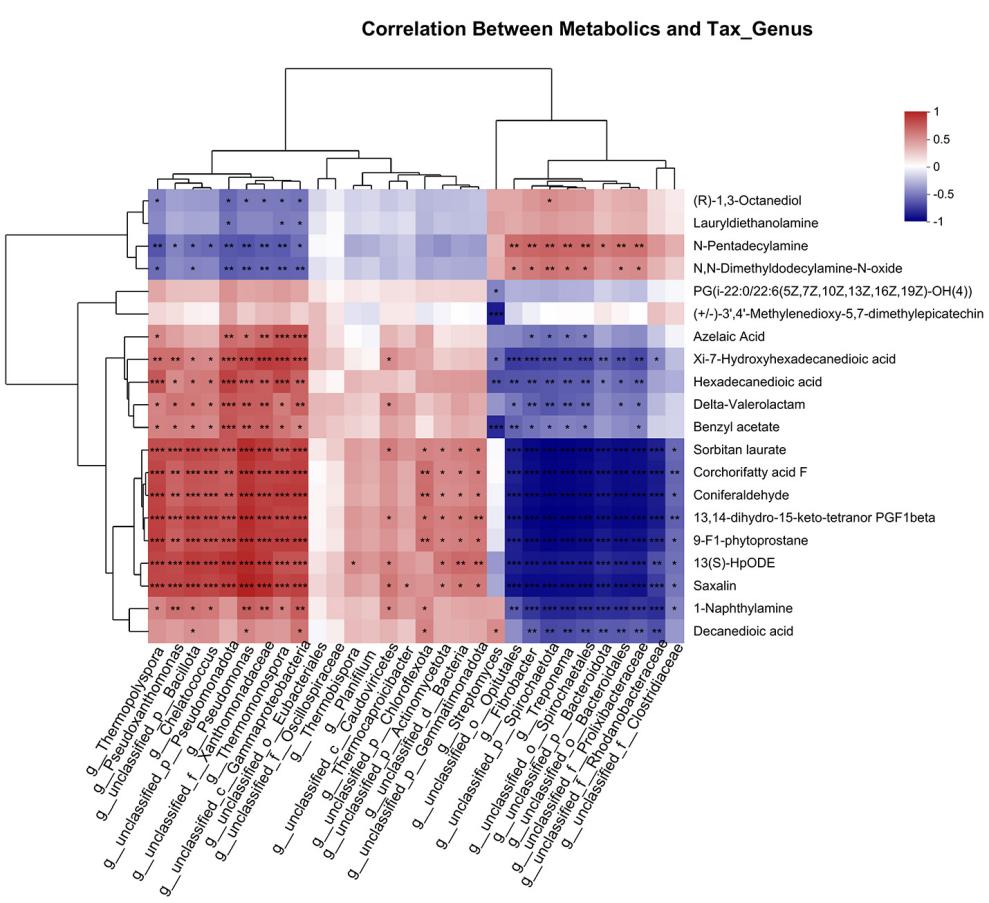


1. Figure. S5 Variation of germinative energy during composting process.


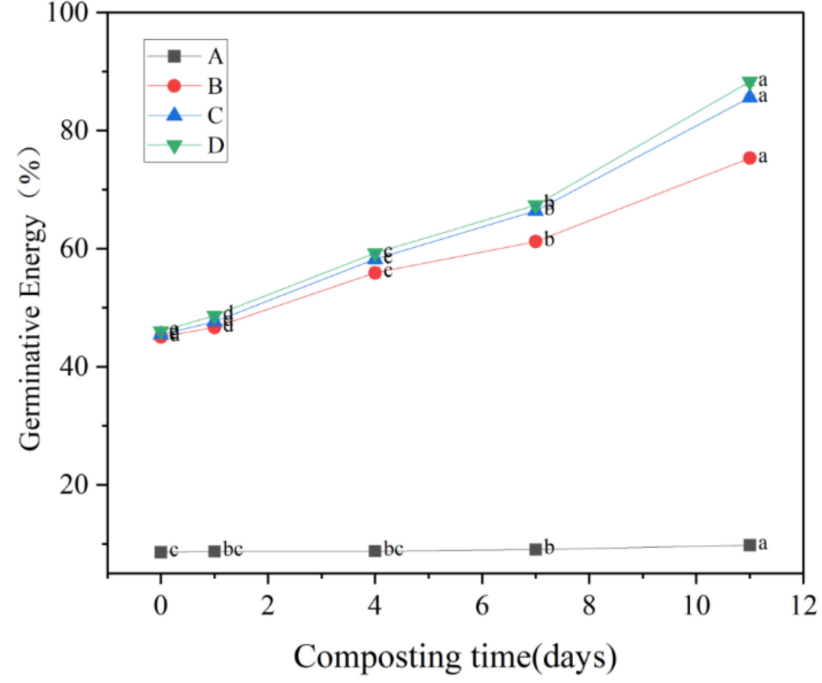


1. Figure. S6 One-way ANOVA for shannon Index


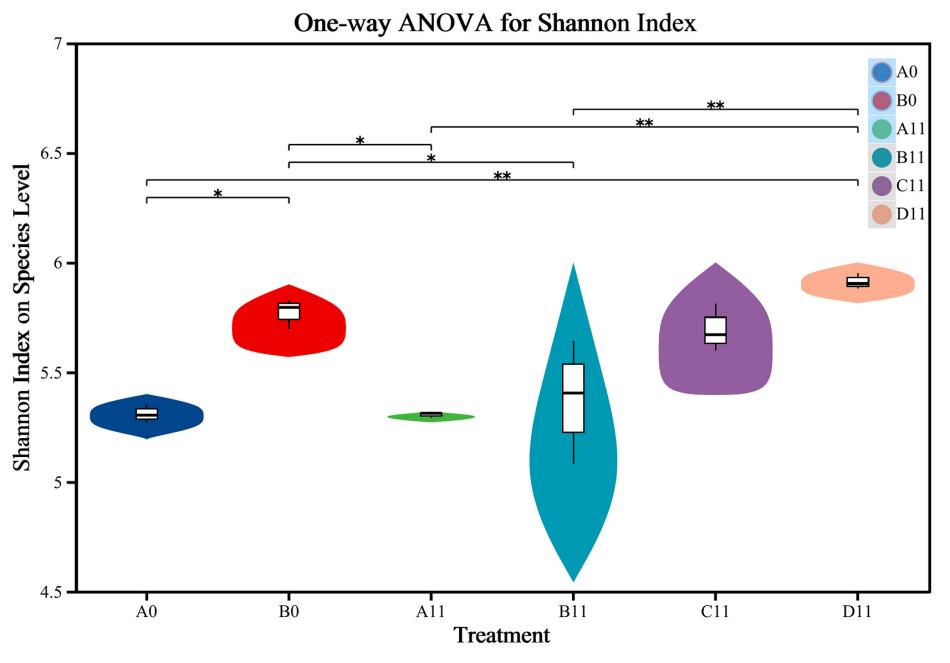
Note: This figure illustrates the significant differences between the two selected groups of samples. The x-axis represents the group names, while the y-axis indicates the index range for each group.

1. Structural equation model showing the potential causal relationships between composting quality (MGE, ARG, temperature), organic carbon (TC, pH), composting properties (TN and CN.ratio) and possible host (bacterial biomass, TK) and potential hosts identified through network analysis.
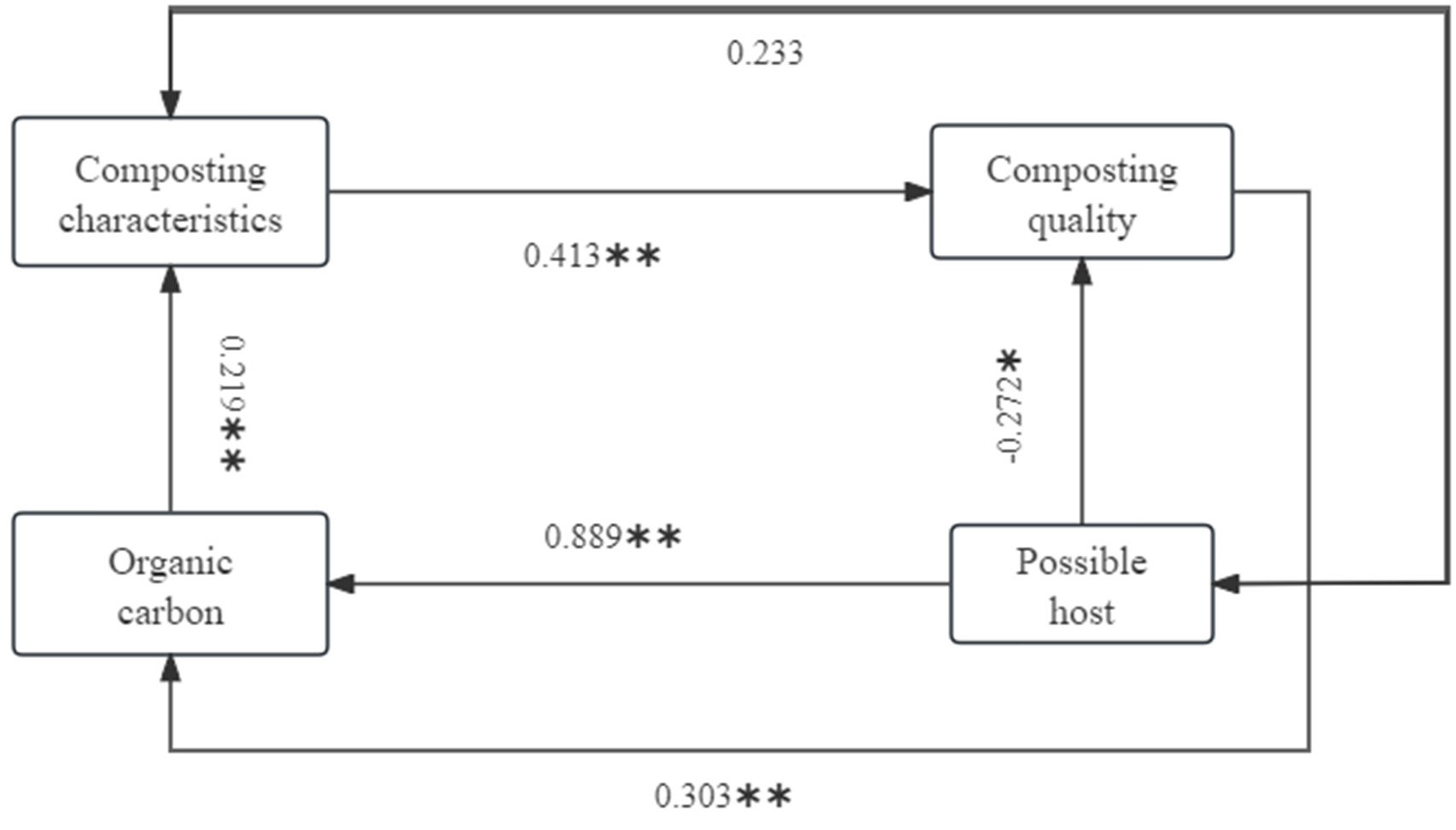


Note:The number and asterisk adjacent to the arrow represent the standardized pathway coefficient and significance level,(*P＜0.05, **P＜0.01, ***P＜0.001).
